# InteGO2: a web tool for measuring and visualizing gene semantic similarities using Gene Ontology

**DOI:** 10.1186/s12864-016-2828-6

**Published:** 2016-08-31

**Authors:** Jiajie Peng, Hongxiang Li, Yongzhuang Liu, Liran Juan, Qinghua Jiang, Yadong Wang, Jin Chen

**Affiliations:** 1 School of Computer Science, Northwestern Polytechnical University, Xi’an, China; 2 School of Computer Science and Technology, Harbin Institute of Technology, Harbin, China; 3 School of Life Science and Technology, Harbin Institute of Technology, Harbin, China; 4 Department of Energy Plant Research Laboratory, Michigan State University, East Lansing, 48824 MI USA; 5 Department of Computer Science and Engineering, Michigan State University, East Lansing, 48824 MI USA

**Keywords:** Gene Ontology, Semantic similarity, Web tool

## Abstract

**Background:**

The Gene Ontology (GO) has been used in high-throughput omics research as a major bioinformatics resource. The hierarchical structure of GO provides users a convenient platform for biological information abstraction and hypothesis testing. Computational methods have been developed to identify functionally similar genes. However, none of the existing measurements take into account all the rich information in GO. Similarly, using these existing methods, web-based applications have been constructed to compute gene functional similarities, and to provide pure text-based outputs. Without a graphical visualization interface, it is difficult for result interpretation.

**Results:**

We present *InteGO2*, a web tool that allows researchers to calculate the GO-based gene semantic similarities using seven widely used GO-based similarity measurements. Also, we provide an integrative measurement that synergistically integrates all the individual measurements to improve the overall performance. Using HTML5 and cytoscape.js, we provide a graphical interface in *InteGO2* to visualize the resulting gene functional association networks.

**Conclusions:**

*InteGO2* is an easy-to-use HTML5 based web tool. With it, researchers can measure gene or gene product functional similarity conveniently, and visualize the network of functional interactions in a graphical interface. *InteGO2* can be accessed via http://mlg.hit.edu.cn:8089/.

**Electronic supplementary material:**

The online version of this article (doi:10.1186/s12864-016-2828-6) contains supplementary material, which is available to authorized users.

## Background

The hierarchical structure and the detailed gene annotation of Gene Ontology (GO) provide biologists a convenient tool to identify enriched gene sets in high-throughput omics-based experiments. In GO, the ontology terms represent biological knowledge and describe functions for genes and gene products. GO consists of three categories, i.e. molecular function (MF), biological process (BP) and cellular component (CC). GO provides rich information as an integrated resource and is convenient to study gene functional similarity [1, 2]. With GO, biologists can quickly test their biological hypotheses and design new experiments [1].

Various computational tools have been developed to identify functionally similar genes or gene products by comparing the annotated GO terms. According to the types of information in GO they use, these methods have been divided into three categories: 1) edge-based measurements, 2) node-based measurements, and 3) hybrid measurements [3, 4]. In the first category, tools are fully dependent on the structure of GO, so that these tools simply treat the terms at the same topological level equally [5]. In the second category, tools consider both the gene annotation and the common ancestors of the target terms. But they neglect the complex topology of GO [6, 7]. In the third category, tools focus on the topological property of the GO structure but neglect the gene annotations [8].

Since none of the existing GO-based gene function similarity measurements can consider all the information in GO (i.e., hierarchical structure, gene annotation, all common ancestors, most informative common parent, *etc.*), we recently proposed two integrative measurements successively to unite the advantage of the existing measures [9, 10]. Our measurements automatically select and integrate seed measurements with a meta-heuristic search based method. In the following text, we briefly introduce our measurements; please refer to the algorithmic details at [9]. Our algorithm has three steps. First, given a background gene set which includes a lot of genes, all their ranked similarity values are pre-calculated with all the selected GO-based semantic similarity measurements (called seed measurements). Second, for every gene pair in user’s input, the most appropriate seed measurements are selected with a grouping method. Finally, we develop a meta-heuristic search model and estimate its parameters by maximizing the distances between distinct EC groups which are manually curated. The algorithm has been tested on MF category, BP category, and protein sequence data. The experimental results indicate that our integrative measurement performs significantly better than the existing measurements.

Various web-based applications have been developed to calculate gene functional similarities based on Gene Ontology. The web-based approach is favorable since users do not need to install tools and maintain the GO data on their computers. The existing web-based GO applications include GossToWeb [11], ProteInOn [12], FunSimMat [13] and G-SESAM [14]. While choosing the best measurement for a specific gene set is critical, none of the aforementioned web-based applications provide a solution. On top of it, most of these tools use the pure text-based format as output. Simply listing gene-to-gene similarity values in a big table neglects the fact that such data visualization is far beyond the direct perception of the human eyes. Biologists face challenges to effectively reduce vast and diverse data into representations that can be interpreted in a biological context. Moreover, there is currently no tools that allow researchers to wander around gene-to-gene associations and make discoveries by following intuition or simple serendipity.

It is desirable to develop an instant interactive web-based application that allows researchers to intuitively explore gene functional similarities and associations, and to visualize the results with an easy-to-use web interface. In this paper, we present *InteGO2*, which, comparing with the existing semantic similarity web tools, has the following major advantages:

- *InteGO2* is an integrative solution toward automatically choosing and weighing gene functional similarity measurements for the user provided gene set.
- *InteGO2* has an easy-to-use HTML5 based web interface. It can effectively visualize the network of genes based on their functional similarities.
- *InteGO2* is available for 98 species and supports 24 kinds of popular Gene ID types.

## Methods

*InteGO2* is a Browser/Server (BS) architecture-based web application. The back end is implemented using Python 2.7 and the web develop framework web.py. MySQL is used for data management. In the front end, Asynchronous JavaScript and XML (AJAX) and JavaScript Object Notation (JSON) are used for efficient data transmission between the browser and server. HTML5 canvas and cytoscape.js [15] are used as the graphics engine for the visualization. The GO annotations of all organisms are downloaded from the GO website (http://www.geneontology.org/) and are updated automatically to ensure that the most recent annotations are used. *InteGO2* embed a gene ID mapping API provided by UniProt website (http://www.uniprot.org/). A user can submit a gene list to web tool using one of the 98 different gene ID types.

## Results and Discussion

*InteGO2* provides a convenient way to calculate and visualize the functional association between genes based on GO. The user guide of InteGO2 is included in Additional file 1. There are two main operations to use *InteGO2*: 1) to submit a gene list and specify parameters, and 2) to visualize and download the gene functional similarities.

### User inputs

**Fig. 1:**
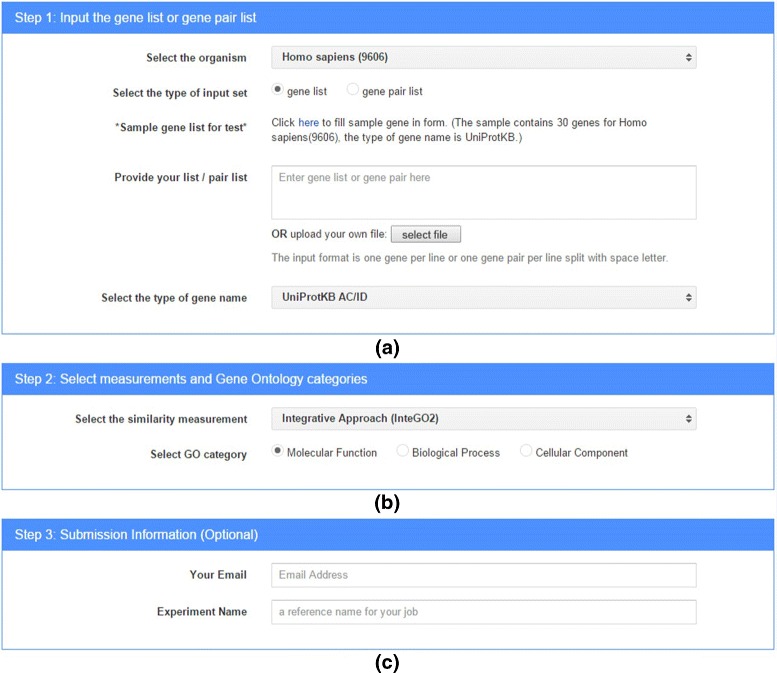
The user input interface of *I* *n* *t* *e* *G* *O*2. The inputs are grouped into three categories: **a** the input genes and related information, **b** choosing similarity measurement and GO category, and **c** user information

The first user interface of *InteGO2* requires inputs in three categories: the input genes and related information (Fig. 1a), choosing similarity measurement and GO category (Fig. 1b), and user information (Fig. 1c). In the first category, a user can input a gene list (or gene pair list) and select the organism and the type of gene name. Currently, 24 organisms are supported (Table 1). Using the ID mapping API from uniProt, we support up to 98 different types of gene IDs belonging to 24 species.

**Table 1:** List of available organisms in InteGO2. Annotated entity count field represents the number of annotated entity. Annotation count field represents the total number of annotations in the annotation file. (Noted that this table may be changed, since the annotation file is updated with the official Gene Ontology website automatically. This table was generated at Feb. 6th, 2015)

	Taxon	Annotated entity	Annotation
		count	count
1	Schizosaccharomyces	5382	39377
	pombe		
2	Aspergillus nidulans	139805	512389
3	Candida albicans	46843	249940
4	Dictyostelium discoideum	8176	62688
5	Saccharomyces cerevisiae	6380	94253
6	Arabidopsis thaliana	30495	239953
7	Rattus norvegicus	20897	352450
8	Gallus gallus	14555	115998
9	Canis lupus familiaris	20342	146570
10	Bos taurus	20418	159295
11	Homo sapiens	45085	455674
12	Sus scrofa	20128	138431
13	Danio rerio	19392	152332
14	Drosophila melanogaster	14614	101879
15	Caenorhabditis elegans	20341	135664
16	Pseudomonas aeruginosa PAO1	1043	1979
17	Leishmania major	644	1905
18	Plasmodium falciparum	2305	5976
19	Trypanosoma brucei	3531	8667
20	Escherichia coli	3770	45976
21	Solanaceae	309	561
22	Dickeya dadantii	124	296
23	Oryza sativa	41141	49292
24	Magnaporthe grisea	11274	27618

In the second category, a user can choose a similarity measurement and a GO category. A recent measurement [5] and six widely-used similarity measurements [6–8, 16–18] are available to choose. Also, we provide an integrative measurement of all the aforementioned approaches [9]. The description of these measurements is in subsection 2.4.

In the third category, a user can leave an email address and the name of the experiment, so that notification will be sent to the user when the job is done. Once all the information is submitted, we validate it for error checking. The validation process checks the format of input genes or gene pairs and all the user specified parameters. The user is notified immediately if any error is found. After that, we calculate the gene-to-gene similarities using the user specified measurement and construct a functional association network.

Note that all the submitted jobs are maintained on the backend server by a job scheduler. Once a job is finished, its job id will be sent to the user who submitted the job, if the user’s email address is provided. If a user does not leave the email address, the user should keep the submission webpage unclosed, so that the experimental results can be displayed on the same webpage. The experimental results will be kept on the back end server for at least two weeks. In addition we also keep the detailed information of the calculation process, such as the number of genes in the input list that cannot be measured because of lack of GO annotations.

### Visualization interface

**Fig. 2:**
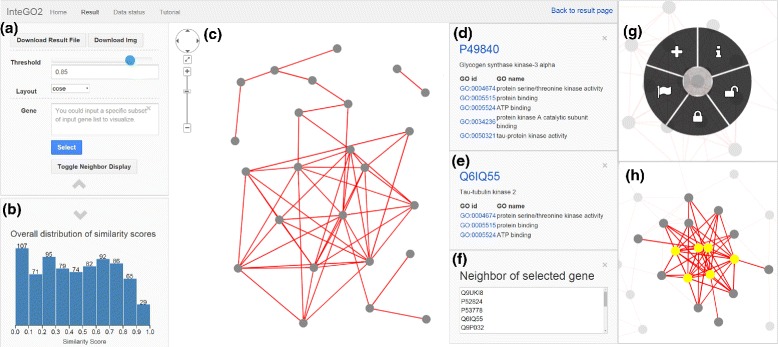
The visualization interface of *I* *n* *t* *e* *G* *O*2 to explore gene functional similarities based on GO. The network is shown in panel (**c**), in which a node represents a gene, and an edge indicates that the similarity score between the two corresponding genes is larger than an edge similarity threshold, which can be changed in panel (**a**). Edge similarity scores distribution shown in panel (**b**) helps users to choose an appropriate threshold. The gene information panel (**d**) and (**e**) show the recently chosen genes and current gene respectively. Panel **f** shows the neighbors of the recently chosen genes. The node operation panel (**g**) allows users to flag, lock or unlock a gene. The selected subnetwork is shown in (**h**)

The visualization interface of *InteGO2* (Fig. 2) shows the resulting gene association network in the center of the web page, in which a node represents a gene, and an edge indicates that the similarity score between the two corresponding genes is greater than an edge similarity threshold. Interactive browsing of the network can be performed conveniently using the mouse: scroll to zoom out or zoom in, left click to select a node, long-left click and drag to move the network, and long-right click a node to activate the node operation panel (Fig. 2g). Using the node operation panel, a user can add the current gene to a gene list of interest shown in panel A, change node color, and lock a node for multiple node operations.

A user can adjust the edge similarity threshold by dragging the threshold bar or inputting a specific value, and the network will change simultaneously (Fig. 2a). To help choose the appropriate edge similarity threshold, the overall edge similarity scores distribution is displayed (Fig. 2b). A user can select different graph layouts for graph visualization (Table 2). A user can also select subnetworks by specifying a gene subgroup (see Fig. 2h).

**Table 2:** The layouts supported in the visualization interface. The six layouts supported in the visualization interface of *InteGO2*

Name	Description
concentric	The concentric layout positions nodes in concentric circles.
	Users could select this layout to put the graph in the middle
	of the explorer.
breadth-first	The breadth-first layout puts nodes in a hierarchy, based
	on a breadth-first traversal of the graph. The hierarchical
	structure of the gene functional association network is
	shown in this layout.
circle	The circle layout puts nodes in a circle. From a circle layout,
	the user could easily find the nodes with high degree and
	low degree.
cose	The cose (Compound Spring Embedder) layout uses a
	force-directed simulation to lay out compound graphs.
	This layout helps the users to find the density region of the
	network.
cola	The cola layout uses a force-directed physics simulation
	with several sophisticated constraints.
grid	The grid layout puts nodes in a well-spaced grid.

The gene information panels (Fig. 2d,e,f) show the recently selected genes, current gene, and the neighbors of the recently selected gene respectively. By clicking a gene ID, a user can fetch more detailed information about the gene from NCBI (www.ncbi.nlm.nih.gov/gene) and the GO term information from Amigo (amigo.geneontology.org).

### An illustrative example of using InteGO2

We use the sample gene list in *InteGO2* website as the example to demonstrate how to use *InteGO*2. First, we set the parameters in Fig. 1a as follows: the organism is *Homo sapiens*, the type of input is “gene list”, and the gene list is the sample gene list provided by the website in the UniProtKB AC/ID format. Second, in Fig. 1b we select “Integrative Approach (InteGO2)” to be the GO similarity measurement and Molecular Function to be the GO category used in the measurement. The parameters in Fig. 1c are optional, but we still enter an email address and provide the experiment name. Finally, we click the “submission” button.

**Fig. 3:**
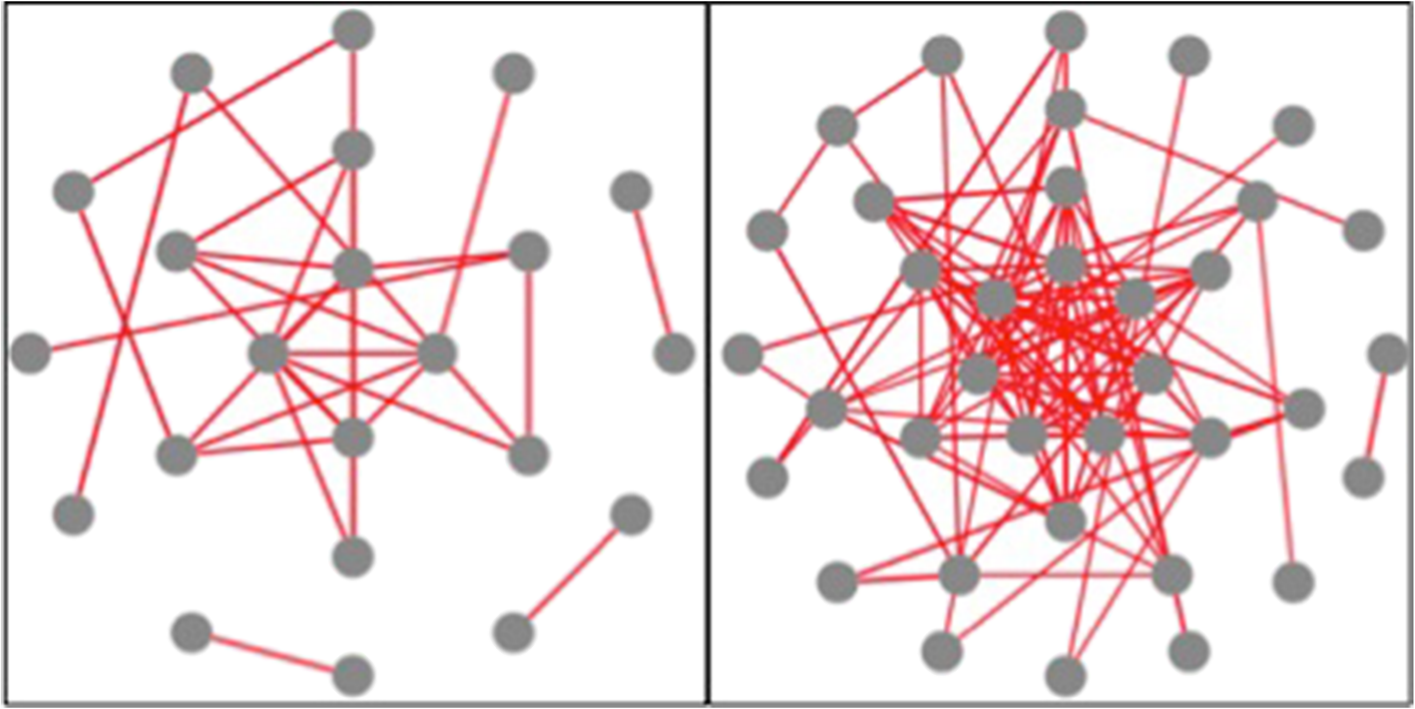
An illustrative example of two networks with different thresholds. An illustrative example of two gene functional association networks with different gene-to-gene similarity thresholds(all the other parameters are the same).The threshold used in the left figure and the right figure are 0.9 and 0.8 respectively

**Fig. 4:**
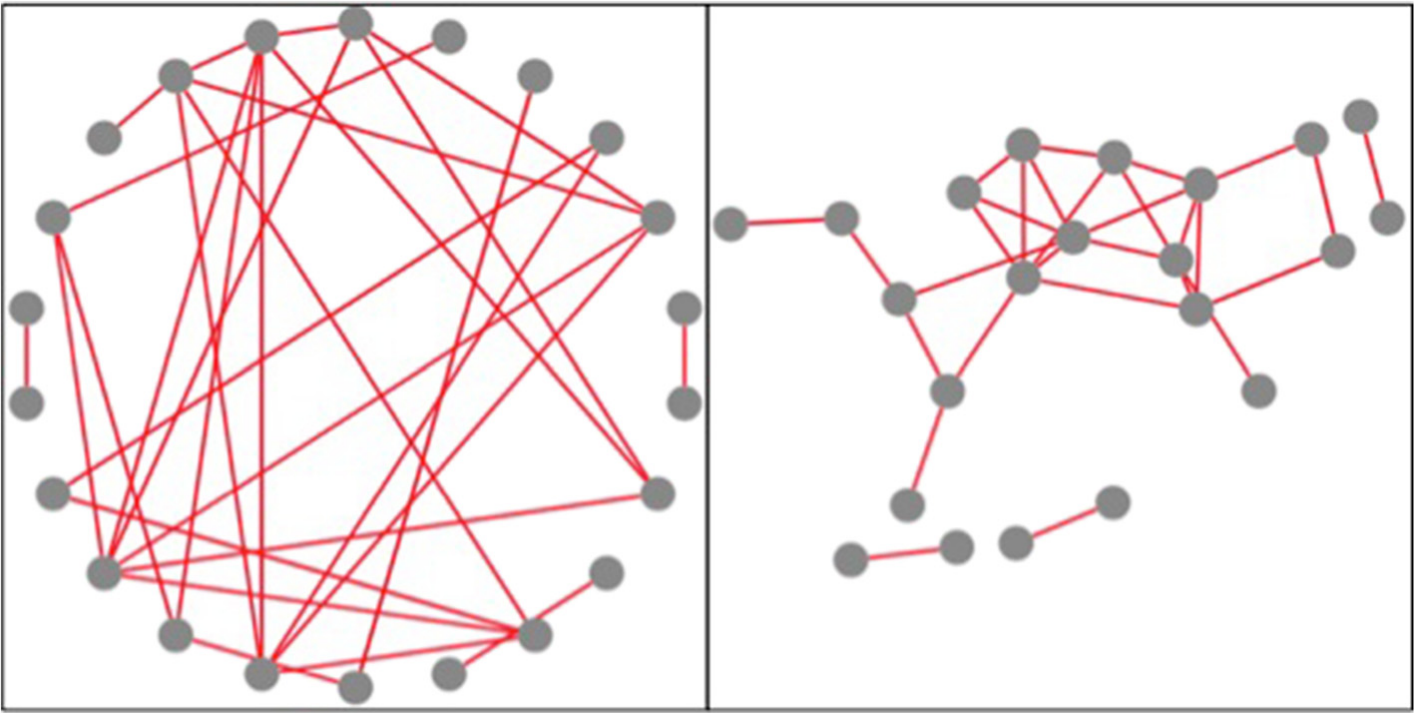
An illustrative example of visualizing a network with two different graph layouts. An illustrative example of visualizing a gene functional association network (left figure in Fig. 3) with two different graph layouts

**Fig. 5:**
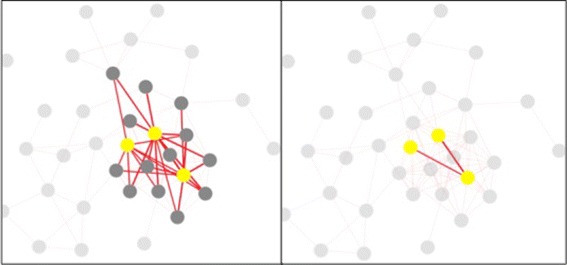
An illustrative example of selecting interested genes to construct subnetworks. The right figure shows three interested genes (*Q*6*I* *Q*55, *P*49840 and *Q*9*B* *Z* *X*2) are selected, and the left figure shows that all the direct neighbors of the interested genes are selected as well

Once our job is finished, we select “Display the visualization of similarity” to view the experimental results using the visualization interface (Fig. 2). By changing the gene-to-gene similarity threshold in Fig. 2a, we generate two gene functional association networks with a different number of nodes and edges (see Fig. 3), and visualize them by selecting two different graph layouts, i.e., concentric and cola (see Fig. 4). Given the gene functional association network in the right figure in Fig. 3, we choose three genes (*Q*6*IQ*55, *P*49840 and *Q*9*BZX*2) as the interested genes, add them into a blank box in Fig. 2a, and click the “select” button. Then the subnetwork that only includes the selected genes is highlighted (see the right figure in Fig. 5). We further add all the neighbor of the selected genes into the highlighted network (see the left figure in Fig. 5) and save it to local hard drive as the final output.

### GO-based semantic similarity measures

Eight GO-based semantic similarity measures are available in our web tool *InteGO*2. In this subsection, we will introduce the eight measurements briefly.

#### 1) Integrative approach (InteGO2)

**Fig. 6:**
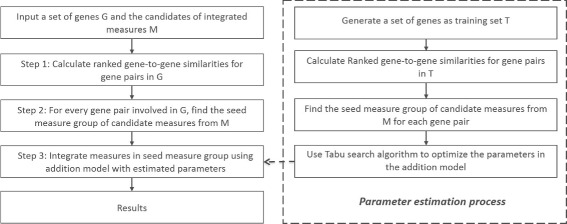
Framework of *InteGO2*. Framework of *I* *n* *t* *e* *G* *O*2 for calculating gene-to-gene similarities for a input gene set (*left*) and for estimating the parameters in the integration model (*right*)

The framework of *InteGO2* is shown in Fig. 6. The whole process contains two parts: one part is gene-to-gene similarity calculation (left) for the input gene set *G*; the other part is model training (right), in which the parameters of *InteGO2* are estimated using a training set *T* by maximizing the distances between distinct EC groups; In *InteGO2*, two key problems are solved, i.e. to choose the most appropriate seed measures for each gene pair from all the candidate measures and to appropriately integrate the selected seed measures.

*InteGO2* is an integrative measure of computing similarity. It automatically selects appropriate seed measures and then integrates them using a meta-heuristic search method [9]. *InteGO*2 has three steps. First, calculate all the similarity scores using all the candidate measures and rank them, resulting in a ranked matrix *M*_*r*_. Second, a grouping process is applied on *M*_*r*_ to identify the common features of all measures, with which we define seed measures for each gene pair, saved in *S*_*can*_. Third, integrate all the measures in *S*_*can*_ with an addition model, in which the weight of each component is estimated by applying a learning process on a training set. Experimental results using ECs and pathways show that *InteGO2* performs better than the existing measures. It also indicates that *InteGO2* is robust against the unavailability of candidate measures. It is noted that an algorithm called *InteGO* was proposed in the previous work to unify different measures [10], which can be considered as a simplified case of *InteGO2*. The new functional association maps generated based on the gene-to-gene similarities based on *InteGO2*, together with the existing biological networks, may provide more biological insights into gene function and regulation.

#### 2) Information content-based (Resnik)

Information Content (IC) of the lowest common ancestor (LCA) is a popular GO term similarity measurement [7], which combines IC and ontology structure. Given a GO term *t*, its IC can be calculated as *IC*(*t*)=−*log*(|*G*_*t*_|/|*G*|), where *G* and *G*_*t*_ represent gene sets annotated to root term and *t* respectively. Given two GO terms *t*_*a*_ and *t*_*b*_, we define *G*_*LCA*_ as gene set annotated to the LCA of *t*_*a*_ and *t*_*b*_. The similarity of GO term *t*_*a*_ and *t*_*b*_ is computed by Eq. 1.

1 $$  Sim_{Resnik}(t_{a}, t_{b}) = IC(LCA) = -log\frac{|G_{LCA}|}{|G|}  $$

#### 3) Normalized information content-based (Schlicker)

Given two GO terms *t*_*a*_ and *t*_*b*_, Schlicker et al. proposed a method to measure their similarity as Eq. 2. The first part of Eq. 2 used IC of *t*_*a*_ and *t*_*b*_ to normalized the IC of their LCA. The second part of Eq. 2 is a weighting score decided by the level of their LCA in GO.

2 $$  Sim_{Schlicker}(t_{a}, t_{b}) = \frac{2 \times IC(LCA)}{IC(t_{a})+IC(t_{b})} \times \left(1 - \frac{|G_{LCA}|}{|G|}\right)  $$

#### 4) Topology information based (Wang)

Different with the gene annotation based measurements, Wang et al. developed a GO topology based method that considers all the ancestor terms [8]. Let *t*_*a*_ and *b* be a GO term and its ancestor term. We define the maximal semantic contribution of the linkages from *t*_*a*_ to *p* as the semantic contribution of *t*_*a*_ to *p*. The similarity of GO term *t*_*a*_ and *t*_*b*_ is defined as follows.

3 $$  Sim_{Wang}(t_{a}, t_{b}) = \frac{\sum_{p \in P_{a} \bigcap P_{b}}{\left(S_{ta,p} + S_{tb,p}\right)}}{\sum_{p \in P_{a}}S_{ta,p} + \sum_{p \in P_{b}}S_{tb,p}}  $$

where *P*_*a*_ and *P*_*b*_ represent the sets of all the ancestors of *t*_*a*_ and *t*_*b*_ respectively.

#### 5) Union information-based (simUI)

Let *g*_1_ and *g*_2_ be two genes. *T*_1_ and *T*_2_ represent the set of GO terms annotation *g*_1_ and *g*_2_. simUI [16] measures similarity as Eq. 4.

4 $$  GeneSim_{simUI}(g_{1}, g_{2}) = \frac{|T_{1} \bigcap T_{2}|}{|T_{1} \bigcup T_{2}|}  $$

#### 6) Graph information content (simGIC)

Combining simUI and Resnik measure, simGIC sums information content (IC) of the terms, not just count the terms [17].

#### 7) Term overlap (TO)

Let *g*_1_, *g*_2_ be two genes and *T*_1_, *T*_2_ be the sets of GO terms annotating *g*_1_, *g*_2_ respectively [18]. TO method computes the similarity score as follows.

5 $$  GeneSim_{TO}(g_{1}, g_{2}) = |T_{1} \bigcap T_{2}|  $$

#### 8) Hybrid relative specificity similarity (HRSS)

Let *t*_*a*_ and *t*_*b*_ be two GO terms. To consider the topological information of GO, relative specificity similarity (RSS) measure the distance from *t*_*a*_, *t*_*b*_ to their closest leaf terms and the distance from *t*_*a*_, *t*_*b*_ to their most recent common ancestor (MRCA). Based on RSS, Wu et al. proposed Hybrid Relative Specificity Similarity (HRSS) employing adapting topology, information content and most informative common ancestor [5]. The similarity score between *t*_*a*_ and *t*_*b*_ is computed by following equations.

6 $$ \begin{aligned} Sim_{HRSS}(t_{a}, t_{b}) = \frac{1}{1+dist(MICA, t_{a})+dist(MICA, t_{b})} \times W \end{aligned}  $$

7 $$ \begin{aligned} W =\frac{IC(root) - IC(MICA)}{IC(root) - IC(MICA) + \frac{IC(t_{a}) - IC(MIL_{a}) + IC(t_{b}) - IC(MIL_{b})}{2}} \end{aligned}  $$

where *root* represents the root term of GO; *MICA* represents the most informative common ancestor of *t*_*a*_ and *t*_*b*_; *MIL*_*a*_ and *MIL*_*b*_ are the most informative child leaf of *t*_*a*_ and *t*_*b*_ respectively; *dist*(*x*,*y*) represents the distance from *x* to *y* in GO; *IC*(*x*) represents the information content (IC) of *x*.

## Conclusions

The Gene Ontology (GO) is a widely used bioinformatics resource. Various methods and web tools have been proposed to compute gene functional similarities based on GO. However, these tools only provide text file or web page includes similarity scores as final output for users, ignoring the appropriate visualization interface for result interpretation.

In this paper, we developed an easy-to-use web tool, named InteGO2, which allows users to conveniently measure gene functional similarity with eight different measures and visualize the resulting gene functional association networks with a web interface. InteGO2 supports up to 98 different of gene IDs belonging to 24 species. The GO data used in InteGO2 tool could be updated automatically to keep consistent with the most recent data from the official website of GO. In summary, InteGO2 is an easy-to-use web tool for researchers to measure and visulize GO-based gene functional similarities.

## Additional file

Additional file 1 User guide. (PDF 608 kb)
