# Erratum to: InteGO2: a web tool for measuring and visualizing gene semantic similarities using Gene Ontology

**DOI:** 10.1186/s12864-017-3651-4

**Published:** 2017-03-28

**Authors:** Jiajie Peng, Hongxiang Li, Yongzhuang Liu, Liran Juan, Qinghua Jiang, Yadong Wang, Jin Chen

**Affiliations:** 1 School of Computer Science, Northwestern Polytechnical University, Xi’an, China; 2 School of Computer Science and Technology, Harbin Institute of Technology, Harbin, China; 3 School of Life Science and Technology, Harbin Institute of Technology, Harbin, China; 4 Department of Energy Plant Research Laboratory, Michigan State University, East Lansing, MI 48824 USA; 5 Department of Computer Science and Engineering, Michigan State University, East Lansing, MI 48824 USA

## Erratum

After the publication of this supplement [1] the URL http://mlg.hit.edu.cn:8089/ceased to work and the new URL of https://mlg.hit.edu.cn/InteGO2 was created to replace it.
