# Supplementary material for: InteGO2: a web tool for measuring and visualizing gene semantic similarities using Gene Ontology
Source: BMC Genomics. 2016 Aug 31;17(Suppl 5):530. doi: 10.1186/s12864-016-2828-6 (PMC5009821; doi:10.1186/s12864-016-2828-6)
Supplement: Additional file 1 — User guide. (PDF 608 kb) [file 12864_2016_2828_MOESM1_ESM.pdf]

Introduction

InteGO2 is an easy-to-use web interface which can be used to calculate and visualize the gene-to-gene functional similarities based on GO.

InteGO2 supplies eight state-of-art gene functional similarity measurements for users, including an integrative approach that automatically choose and weigh appropriate measurements for the input genes. Currently, 24 organisms are available. The GO annotations of all organisms are downloaded from the GO website and are updated automatically to ensure that the most recent annotations are used.

There are two main processes to use InteGO2, including:

- input the parameters and gene list;
- visualize and download the result of gene functional similarities.

The input interface of InteGO2

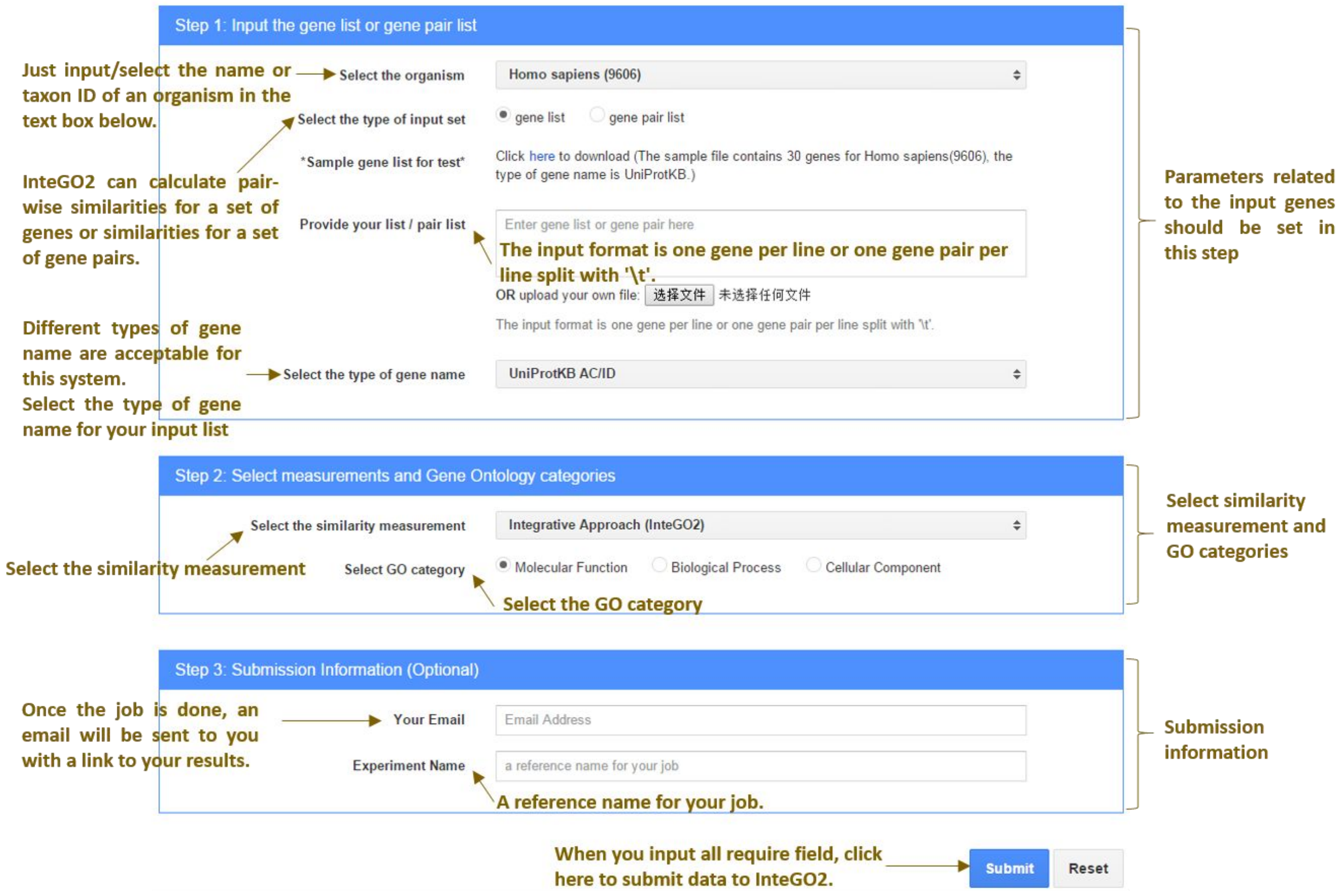

Fig.1 the input interface of InteGO2

After the user has submitted his/her data, InteGO2 validates the input for errors. The validation checks the format of input gene or gene pair list and all the options and parameters uploaded. The user is notified immediately, if any error is found. After the input data passed the validation

process, InteGO2 starts to calculate the gene-to-gene similarities. The user could download and visualize the results, once the calculation process is done. At the same time, a link to the results and visualization interface is emailed to the user.

Download and visualize Similarity result

Once the calculation process is done, the user could choose to visualize the result or download it (see following page). The user also could visit the previous results saved on InteGO2 server via the runid.

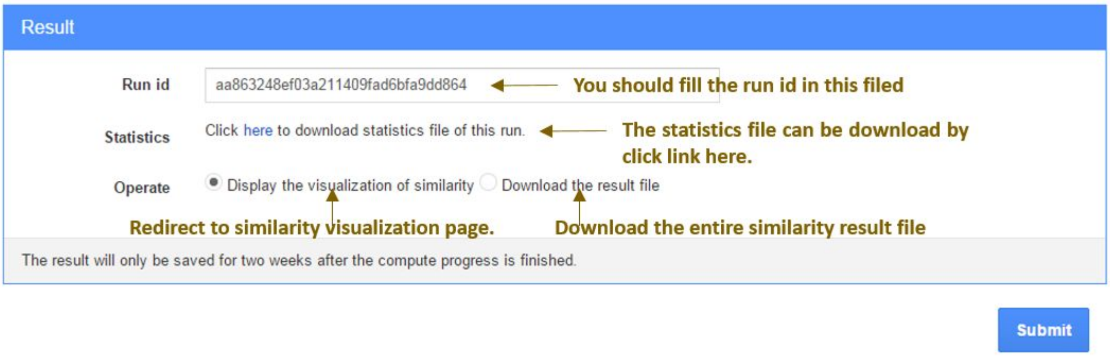

Fig.2 Result page of InteGO2

Following is the visualization interface of InteGO2.

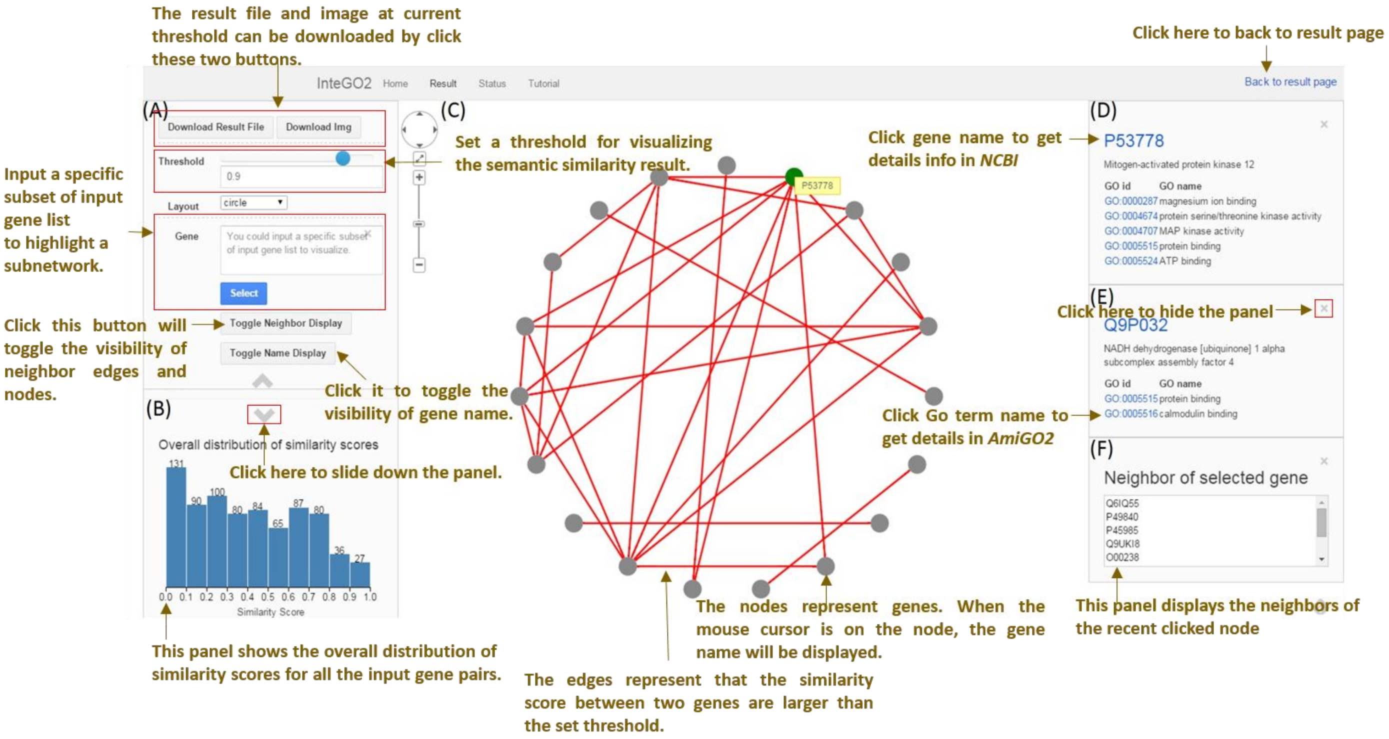

Fig.3 Visualization page

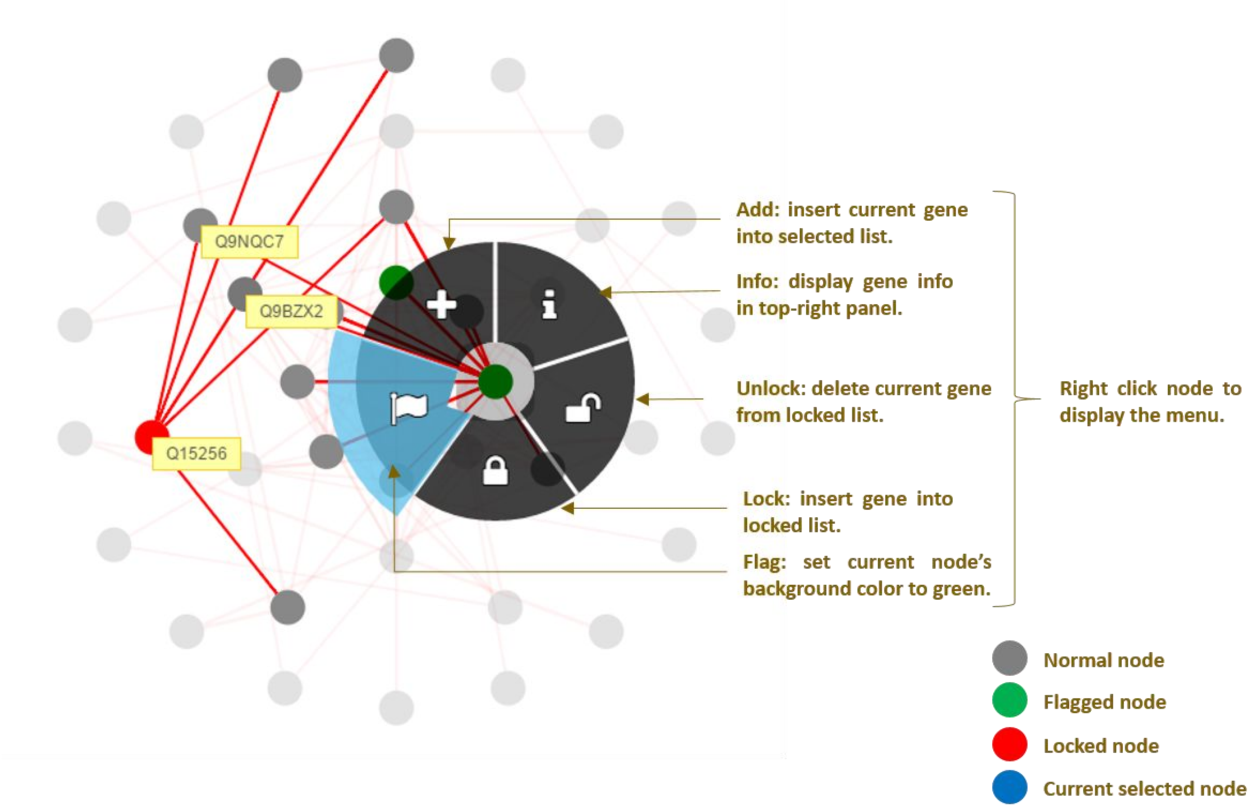

Fig.4 More detail about result visualization page

Note: The similarity result will only be saved for two weeks after the compute progress is finished.
